# Anorectal manometry after rectal cancer surgery: physiological insights, clinical interpretation, and limitations

**DOI:** 10.1007/s10151-026-03400-x

**Published:** 2026-08-07

**Authors:** K. L. Park, D. K. Sohn

**Affiliations:** Center for Colorectal Cancer, National Cancer Center, Ilsan-Ro 323, Ilsan-Dong-Gu, Goyang, Republic of Korea

**Keywords:** Anorectal manometry, Rectal neoplasms, Quality of life, Low anterior resection syndrome, Anorectal function

## Abstract

Anorectal manometry (ARM) provides objective physiological information on anal sphincter function, rectal or neorectal sensation, reservoir capacity, and anorectal reflexes after rectal cancer surgery. In rectal cancer surgery, ARM has been used to evaluate functional changes after sphincter-preserving procedures, reconstruction with colonic J-pouch, neoadjuvant radiotherapy, and postoperative bowel dysfunction, including low anterior resection syndrome (LARS). This review summarizes the clinical applications of ARM in rectal cancer surgery, with emphasis on historical and contemporary manometric parameters, their relationship with patient-reported outcomes, and their limitations in postoperative functional assessment. Although several studies have reported associations between manometric findings and bowel dysfunction, the clinical interpretation of ARM remains limited by heterogeneity in measurement protocols, equipment dependency, timing of assessment, and variable correlation with quality-of-life measures. Recent advances, including high-resolution manometry and standardized testing protocols, may improve the reliability and clinical utility of ARM. ARM may provide adjunctive physiological information in selected patients and research settings, but current evidence does not support its use as a stand-alone diagnostic test for LARS or as a routine treatment-decision tool.

## Introduction

The evolution of functional anorectal manometry (ARM) from the early twentieth century to the present represents a significant progression in medical diagnostics and treatment. Initially, medical understanding of intestinal motility was limited to visualizing the passage of intraluminal balloons using radiography [1], revealing that the alimentary tract combines segmentation and peristalsis. The development of a hydraulic intraluminal recording system by Arndorfer et al. [2] in 1977 marked a significant breakthrough, introducing a quantitative approach to understanding gastrointestinal function. Arndorfer et al. devised the apparatus based on the hypothesis that flow (*F*) across the alimentary canal is proportional to the difference between the pressure (P1) in a fixed chamber and the measured luminal pressure (P2) (*F* ∝ P1 − P2). Providing a benchmark on a pressure graph, this apparatus enabled quantitative measurement and comparative presentation of anorectal function and became the prototype for manometry. Moreover, the authors’ attempt to statistically validate human esophageal motility accelerated the dissemination of functional manometry in clinical research [3]. These quantitative measurements have been particularly impactful in pediatric conditions, such as achalasia and hypertrophic pyloric stenosis, where evaluating clinical symptoms through conversation or patient feedback is challenging. Similarly, anorectal manometry has been used in the evaluation of Hirschsprung disease by assessing the rectoanal inhibitory reflex (RAIR). The absence of RAIR supports the diagnosis by reflecting failure of reflex relaxation of the internal anal sphincter in response to rectal distension [4]. The aforementioned conditions can be diagnosed using plain radiography; however, manometry remains the gold standard owing to its ability to distinguish subtypes of each condition for which different treatment approaches are indicated [5].

The recent introduction of ARM in rectal cancer surgery has introduced novel dimensions to the field. Unlike its traditional use for evaluating intestinal motility, ARM in this setting focuses on anal sphincter function, anorectal reflexes, and rectal or neorectal sensation and reservoir function, depending on the surgical anatomy. When broadly classified, ARM is used to evaluate the following: (1) anorectal function according to the surgical method, (2) anorectal function decline due to neoadjuvant radiation, and (3) postoperative quality of life (QOL) after rectal surgery. Certain limitations in ARM measurements have led to debates on its reliability, necessitating further research and standardization [6]; nevertheless, ARM can provide objective physiological data on anal sphincter pressure, rectal or neorectal sensation, and reservoir function. These measurements may be useful as adjunctive information in selected clinical and research settings, although their interpretation requires caution because of heterogeneity in measurement protocols and variable correlation with patient-reported symptoms.

In this study, we aimed to provide an overview of ARM measurements and review cases in major clinical studies, focusing on the utilized parameters. In addition, we aimed to describe recent research trends, ARM limitations, and future directions. The history of ARM from its early roots in visualizing intestinal movement to its current applications in diverse medical and surgical fields is a testament to the relentless pursuit of improving diagnostic accuracy and highlights the importance of further medical innovations.

## Literature search strategy

This narrative review was based on a literature search of PubMed, Embase, and Web of Science for articles published up to December 2025. The search terms included combinations of “anorectal manometry,” “rectal cancer,” “low anterior resection,” “low anterior resection syndrome,” “fecal incontinence,” “quality of life,” “radiotherapy,” “colonic J-pouch,” “high-resolution manometry,” and “pelvic floor rehabilitation.” Studies were selected if they addressed anorectal or neo-anorectal physiological assessment in patients undergoing rectal cancer treatment, including sphincter-preserving surgery, reconstruction, neoadjuvant or adjuvant radiotherapy, stoma reversal, and postoperative bowel dysfunction. Additional relevant articles were identified from the reference lists of selected publications. Because of heterogeneity in study design, manometry systems, measurement timing, and reported outcomes, a formal meta-analysis was not performed.

## Measurement of functional ARM

**Fig. 1:**
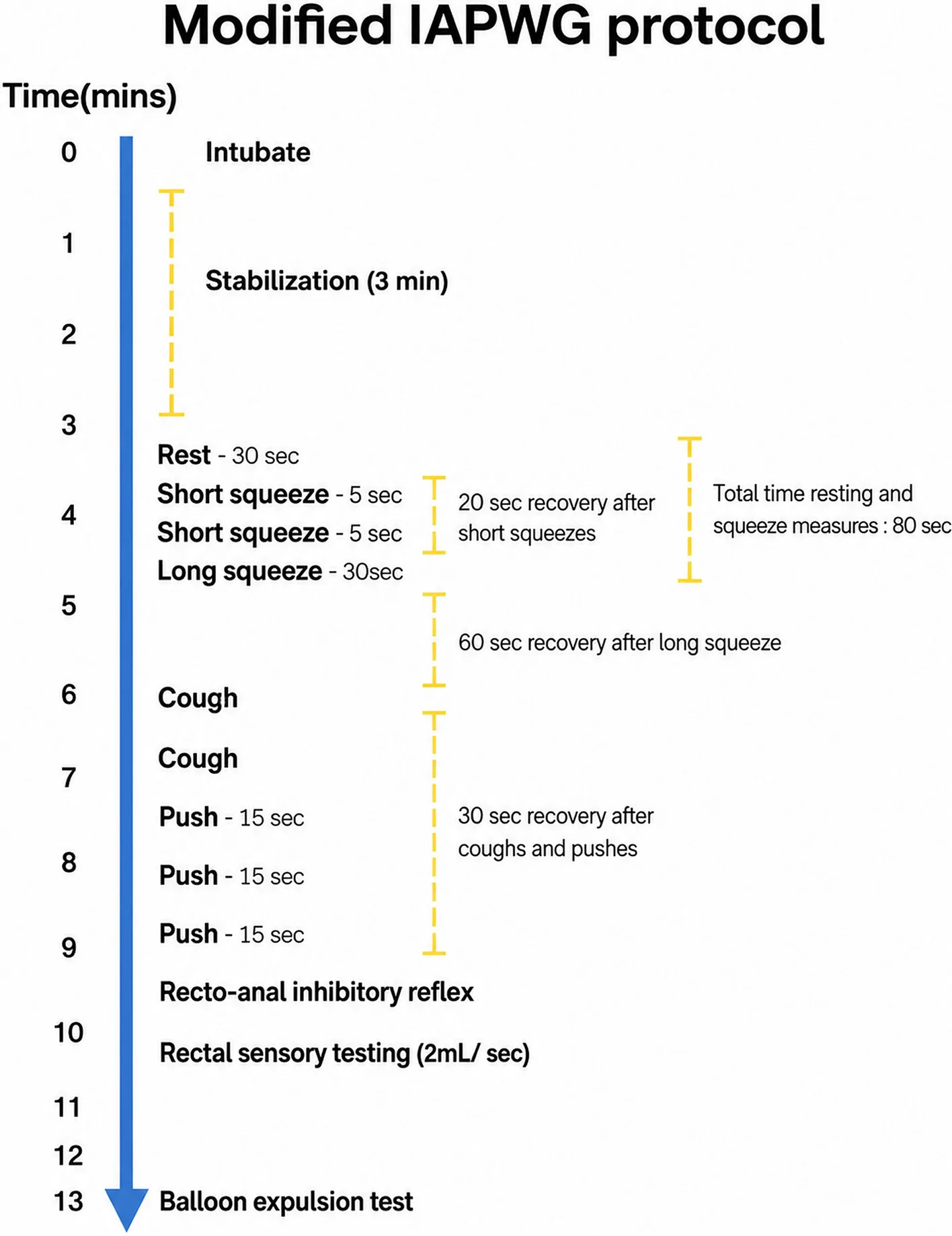
Schematic of functional ARM measurement by the IAPWG protocol. *ARM* anorectal manometry, *IAPWG* International Anorectal Physiology Working Group

The measurement method should be standardized and precisely performed without errors to obtain accurate results from tests and effectively apply them in clinical practice. The International Anorectal Physiology Working Group (IAPWG) [7]—the first worldwide consensus group—launched and announced a standardized ARM protocol in 2020 (Fig. 1). The measurement of functional ARM comprises the following steps: (1) stabilization period (i.e., following catheter insertion, 3 min should be observed to relax the anal sphincter tone and zero the catheter sensor probes); (2) pressure manometry stage, which involves measuring parameters such as resting pressure and squeeze pressure; and (3) the volumetric manometry stage, which involves measuring rectal sensory parameters and the rectoanal inhibitory reflex (RAIR).

### Pressure parameters

Pressure manometry data are typically collected during the first stage of the manometry examination using linear catheters containing pressure sensors. During this stage, the involuntary tones of the anal sphincters in the resting state or during coughing are recorded. Afterward, voluntary sphincter tones during squeezing and pushing efforts are measured.

#### Resting pressure

At the initial stage of manometry examination, patients are asked to achieve a state of complete relaxation. This step involves measuring the basal anal tone at rest, and recording measurements for at least 60 s is recommended. Eight pressure probes measure the anal sphincter tone: the internal sphincter accounts for 55%, the external sphincter for 35%, and the hemorrhoidal plexus for the remaining 15% [8]. The average value obtained during this period is defined as the mean resting pressure (mRP), and the maximum value is referred to as the maximum resting pressure (MaxRP). The mRP ranges from 56 to  88 mmHg in healthy individuals, and normal aging, female sex, and multiparity are associated with a lower mRP [9]. Prichard et al. [10] reported that decreased mRP has 51% sensitivity and 70% specificity for internal sphincteric damage. These findings are significant in rectal cancer research because physical stimulation of the internal sphincter by rectal tumors may impair sphincter function. Moreover, inevitable pudendal nerve injuries due to total mesorectal excision (TME) or radiotherapy may cause anal sphincter dysfunction.

#### Squeeze pressure

Conventionally, patients are instructed to squeeze their anal sphincter after measuring the resting pressure. Three squeezes are performed for 5 s, with a 30-s recovery interval. The average and maximum values measured at this time are defined as the mean squeeze pressure (mSP) and maximum squeeze pressure (MaxSP), respectively. MaxSP ranges from 167 to 247 mmHg and is decreased in patients aged > 65, those with female sex, and multiparous women since voluntary contractile force is proportional to lean muscle mass [11]. Squeeze pressure is reported to have 79% specificity for predicting external sphincter or puborectalis injury or patulous anal canal [10] and has been utilized in studies on benign anal disease.

#### Functional anal canal length

High-resolution manometry with up to 36 catheters simultaneously positioned across the anal canal has enabled the tomographic analysis of segments with consistent pressure, allowing the measurement of anal canal length. Functional anal canal length is defined as the area exceeding the resting pressure by at least 5 mmHg. The length of the functional anal canal is 3.6–4.2 cm in healthy individuals, decreasing with the presence of fecal incontinence and normal aging [12].

### Volumetric parameters (rectal sensory testing)

After completing the pressure manometry measurement, the examiner typically connects a new sensor catheter with an attached balloon to the manometry device. The sequentially measured variables as the balloon volume increases from zero are termed “volumetric parameters” (rectal sensitivity testing). After rectal cancer surgery, these measurements may reflect rectal or neorectal sensation and reservoir function, depending on the type of resection and reconstruction. Therefore, volumetric parameters should not be interpreted as measurements of normal rectal physiology in all postoperative patients, but rather in the context of the remaining rectum, neorectum, or reconstructed bowel segment.

#### Initial sensation volume (IniSense)

Inisense represents the volume at which the patient first perceives the “desire to defecate” as the catheter balloon inflates. The normal range is 32–44 mL in the healthy population. Inisense, which reflects anorectal hyper/hypoactivity, tends to diminish with the presence of fecal incontinence and increases with multiparity and normal aging.

#### Urge volume (UrgeV)

UrgeV is defined as the volume at which the patient first perceives the “urge to defecate” after IniSense as the balloon continues to inflate. UrgeV ranges between 77 and 98 mL in healthy populations [9]. UrgeV, along with RAIR and IniSense, serves as an indicator of intestinal sensitivity and has diagnostic value in Hirschsprung disease and irritable bowel syndrome.

#### Maximum tolerable volume (MaxTV)

MaxTV is the volume at which the balloon expands over UrgeV, and examinees subjectively experience maximum discomfort. The literature recommends not inflating the catheter balloon to more than 250 mL as a safety precaution to prevent excessive luminal distension [11]. MaxTV ranges from approximately 140 to 159 mL in healthy populations, although values vary according to the measurement protocol, catheter system, balloon characteristics, age, sex, and prior pelvic surgery [9, 11]. Compliance (CO, mL/mmHg), derived from the pressure–volume relationship recorded during MaxTV measurement, aids in intuitively understanding the status of the rectal wall. Rectal CO decreases after pelvic radiotherapy and in patients with idiopathic bowel inflammation or chronic ischemia. In contrast, increased CO indicates a plicated rectal wall in patients with functional constipation. Collectively, volumetric parameters (IniSense, UrgeV, MaxTV, and CO) are associated with visceral hypersensitivity, such as allodynia, hyperalgesia, irritable bowel syndrome, and psychological bowel function disorder [6, 13].

#### Rectoanal inhibitory Reflex (RAIR)

RAIR refers to reflex relaxation of the internal anal sphincter in response to rectal distension [14]. In ARM, RAIR is commonly assessed by stepwise inflation of a rectal balloon, and the reflex response generally varies according to the degree of rectal distension. Although RAIR is diagnostically important in conditions such as Hirschsprung disease, its interpretation after rectal cancer surgery is more complex because rectal resection, neorectal reconstruction, radiotherapy, anastomotic height, and altered reservoir function may all influence the reflex response. In rectal cancer research, RAIR has been reported as a possible indicator of functional impairment after rectal surgery [15]; however, these findings have not been consistently replicated. Therefore, RAIR should be interpreted cautiously and should not be used alone to assess postoperative bowel dysfunction.

### Vector parameters

#### High-pressure zone (HPZ)

HPZ refers to the length of the high-pressure segments, which reflect the contractile portion between the puborectalis and the external sphincter. Conventionally, functional ARM measures intraluminal pressure using eight linear probes installed on a single catheter. A puller removed from the patient’s body moves the positions of the linear probes at a steady pace to compute the profile of the entire anal canal. The HPZ technique converts the radial pressure asymmetry caused by the catheter height into volume to obtain a three-dimensional blueprint of the entire anal canal [16]. Vector volumetry has the limitation of requiring an additional three-dimensional manometry catheter. Nevertheless, vector volumetry may continue to be utilized in future research, considering that most of the latest versions of ARM equipment aiming to correct inter-examiner discrepancies are equipped with this parameter [13].

## Studies using ARM in rectal cancer treatment

### Application of ARM in assessing diverse surgical options for low rectal cancers

The first use of functional ARM in rectal cancer treatment focused on pelvic floor dysfunction occurring after sphincter-sparing rectal cancer surgery. Most of these studies adopted manometry to quantitatively assess anorectal sphincter capacity, hypothesizing that the surgical approach may impair anorectal defecatory function. Background evidence suggested that TME for low rectal cancer physically impinges on the internal anal sphincter and the sphincter innervation of the pudendal nerve. The studies mentioned below quantitatively and statistically reported decreased anorectal function postoperatively and hold clinical significance as some of the first attempts to quantitatively present the consequences of the surgical methodology. Table 1 summarizes the manometric parameters utilized in studies that analyzed the relationship between anorectal function and surgical approach for low rectal cancer. The validated variables, with results replicated in successive studies, were resting pressure, MaxTV, and anal canal length.

**Table 1:** Manometric parameters of previous studies analyzing anorectal dysfunction after surgery for low rectal cancer

Authors	Object of comparison	F/U interval	Parameters of statistical significance	Interpretation of results
1992 O’Riordain [15] *N* = 46	LAR, preoperative value vs LAR, postoperative value	1 year	mRP RAIR	Patients who received LAR showed a decrease in mRP and RAIR compared to the preoperative value 1 year after surgery
		2 years	mRP RAIR	Patients who received LAR showed a decrease in mRP and RAIR compared to the preoperative value 2 years after surgery
1994 Schiessel [17] *N* = 38	ISR, preoperative value vs ISR, postoperative value	2 years	mRP ACL	Patients who received ISR showed a decrease in mRP and a shorter ACL compared to preoperative value 2 years after surgery
2003 Rasmussen [19] *N* = 43	*(Among patients who received LAR)* Fecal incontinence (−) vs Fecal incontinence (+)	1 year	MaxTV CO	Patients who underwent LAR and complained of fecal incontinence showed a decreased MaxTV and CO compared to patients who underwent LAR but did not complain of fecal incontinence 1 year after surgery
2003 Matzel [20] *N* = 33	AR vs. LAR vs. ISR	6 months	mRP MaxRP UrgeV	Patients who underwent LAR and ISR showed decreased mRP and MaxRP compared to patients who underwent AR 6 months after surgery. Patients who underwent ISR showed a decreased UrgeV compared to patients who underwent AR 6 months after surgery
2009 Koda [18] *N* = 64	AR vs. LAR vs. ISR	1 year	ACL, resting ACL, squeeze HPZ	Patients who underwent LAR and ISR showed a shorter ACL and HPZ compared to patients underwent AR 1 year after surgery

*AR* anterior resection, *LAR* low anterior resection, *ISR* intersphincteric resection, *mRP* mean resting pressure, *MaxRP* maximum resting pressure, *RAIR* rectoanal inhibitory reflex, *ACL* anal canal length, *MaxTV* maximum tolerable volume, *CO* compliance, *UrgeV* urge volume, *HPZ* high-pressure zone

In 1992, O’Riordain et al. [15] described incontinence after low anterior resection (LAR) using ARM and presented a pressure profile in the feces-free rectum using 2 years of follow-up data, demonstrating significant decreases in mRP and the RAIR. In 1994, Schiessel et al. [17] reported the pressure manometry profile of patients who underwent intersphincteric resection (ISR), with a design similar to that of the study by O’Riordain et al. Both studies found that the squeeze pressure, which is conditioned by external sphincter activity, did not decrease as significantly as expected after surgery. This was interpreted to reflect the surgical techniques that spare the somatic nerve innervating the external sphincter in the anatomical plane of the TME.

Moreover, Schiessel reported a significantly shortened anal canal length postoperatively. Attempts to correlate shortened anal canal length with anorectal dysfunction continued until 2009 when Koda et al. [18] further developed a variable HPZ. Koda et al. reported a shorter anal canal length and HPZ as anastomosis height decreased in patients who underwent different surgical procedures, including anterior resection, LAR, and ISR.

Rasmussen et al. [19] published a study with a unique design in 2003, comparing the manometry profile according to the presence or absence of fecal incontinence in patients who underwent LAR. This design initiated a more in-depth investigation of the relationship between numerical ARM data and subjective symptoms reported by patients. The authors concluded that the volumetric parameters MaxTV and CO better reflected the patients’ subjective symptoms. Furthermore, Matzel et al. [20] demonstrated diminishing resting pressures and UrgeV parameters as anastomosis height decreased, with a statistically significant QOL impairment. These two studies aimed to evaluate rectoanal function by linking QOL data, a subjective variable, and manometry data, a numerical variable, suggesting a direction for modern research.

### Application of volumetric ARM in J-pouch reconstruction surgeries

The J-pouch, first introduced in 1986 [21], is a surgical technique designed to expand the reservoir capacity of the neorectum and remains a controversial method. A series of randomized clinical trials (RCTs) have attempted to quantify this construct, demonstrating unexpected ARM outcomes. Ho et al. [22] published an RCT in 1996 comparing conventional coloanal anastomosis with the J-pouch. This study hypothesized that the expanded neorectum would structurally enhance the volumetric ARM parameters compared to conventional surgery. The J-pouch showed superiority for patients’ QOL; however, no intended volumetric benefit from this novel surgical design was observed. Furthermore, significantly diminished pressure ARM profiles were observed following the J-pouch. Subsequent studies [23–25] reported improvements in QOL by the J-pouch; however, reproducibility regarding ARM scores was not observed with the secured J-pouch volume. Similarly, subsequent large-scale RCTs using only QOL questionnaires reported the efficacy of the J-pouch [26, 27]. As shown in this context, despite repeated confirmation of improved QOL with the J-pouch, discrepancies with ARM data have been identified. This case underscores the necessity for the standardization of ARM measurements to solidify the method’s utility in clinical research. Table 2 lists the manometry data from studies analyzing the reservoir expansion effect of the J-pouch.

**Table 2:** Manometry data of studies analyzing the reservoir expansion effect of the J-pouch

Authors	Object of comparison	F/U interval	Parameters of statistical significance	Interpretation of results
1996 Ho [22] N:33	Straight LAR vs LAR with J-pouch	1 year	mRP MaxSP	Patients who underwent LAR with J-pouch reconstruction showed decreased mRP and MaxSP compared to patients who underwent LAR alone 1 year after surgery
1997 Hallböök [23] *N* = 46	Straight LAR vs LAR with J-pouch	1 year	Neorectal volume CO	Patients who underwent LAR with J-pouch reconstruction showed an increased neorectal volume and CO compared to patients who underwent LAR alone 1 year after surgery
1998 Joo [25] *N* = 83	Straight LAR vs LAR with J-pouch	6 months	No significance	No significant manometric differences were found between patients who underwent LAR alone with those who underwent LAR with J-pouch 6 months after surgery
		1 year	CO	Patients who underwent LAR with J-pouch showed increased CO 1 year after surgery
2002 Furst [24] *N* = 74	Straight LAR vs LAR with J-pouch	6 months	No significance	No significant manometric differences were found between patients who underwent LAR alone with those who underwent LAR with J-pouch 6 months after surgery

*LAR* low anterior resection, *mRP* mean resting pressure, *MaxSP* maximum squeeze pressure, *CO* compliance

### Anorectal manometry for safety assessment of preoperative radiotherapy

In the 2000s, the popularity of radiotherapy surged in the field of rectal cancer treatment. Beginning with the 1995 Swedish trial [28], radiotherapy demonstrated exceptional potential for local control and downstaging [29]. This influence significantly affected the preferred oncological approach to treating middle and lower rectal cancers. This novel modality continued to advance as scholars began testing treatment timing, with Sauer et al. [30] demonstrating survival benefits of neoadjuvant radiotherapy over adjuvant treatment. In 2003, Ammann et al. [31] first compared a neoadjuvant radiotherapy group with a surgery-only group and reported anal sphincter dysfunction with decreased mRP and MaxTV due to radiotherapy. Subsequently, Pollack et al. [32] conducted a 14-year follow-up investigation of the Swedish trial cohort. Their results revealed reduced QOL and manometric parameters, including MaxRP and MaxSP, after neoadjuvant radiotherapy. They observed similar manometric changes upon analyzing patients’ discomfort (i.e., fecal incontinence) as a variable independent of radiotherapy status. This suggests that interpretative differences may exist regarding the toxic effect of radiotherapy on anorectal function. During the same period, two national registry-based large cohort questionnaire studies [33, 34] provided evidence of radiotherapy-induced anorectal function impairment. To the best of our knowledge, Ihnát et al. [35] most recently reported a radiotherapy-induced decline in manometric parameters (mRP, IniSense, UrgeV, CO, and MaxTV) in 2018.

Nevertheless, in recent years, the debate on radiotherapy toxicity has begun to adopt a different perspective. In 2010, the Canada Clinical Trials Group C016 [36] published a national registry-based RCT of 1350 participants. This study compared patients who received preoperative radiation therapy and those who received selective postoperative chemoradiation with 2 years of follow-up, revealing that radiotherapy did induce a minor defecatory problem, which resolved after 1 year. Male sexual dysfunction was the only radiotherapy-induced toxic effect that appeared over the 2 years. Similarly, in 2010, Canda et al. [37] reported a significant decrease in mRP in the surgery-only and preoperative radiotherapy groups. They concluded that while radiotherapy-induced anorectal dysfunction was evident, neoadjuvant radiotherapy remains advantageous when weighing its risks against its oncological benefits, considering reports of long-term recovery. Researchers have interpreted this shift in research results as a technological advancement in radiotherapy [38]. The manometric parameters of the studies that analyzed the relationship between anorectal function and preoperative radiotherapy are listed in Table 3.

**Table 3:** Manometric parameters of studies analyzing the relationship between anorectal function and preoperative radiotherapy

Authors	Object of comparison	F/U interval	Parameters of statistical significance	Interpretation of results
2003 Ammann [31] *N* = 50	LAR only vs NART with LAR	1 year	mRP MaxTV	Patients who underwent NART with LAR showed decreased mRP and MaxTV compared to patients who underwent only LAR 1 year after surgery
2007 Pollack [32] *N* = 64	LAR only vs NART with LAR	14 years	MaxRP MaxSP	Patients who underwent NART with LAR showed decreased MaxRP and MaxSP compared to patients who underwent only LAR 14 years after surgery
2010 Canda [37] *N* = 57	LAR only vs NART with LAR	1 year	MaxSP	Patients who underwent NART with LAR showed decreased MaxSP compared to patients who underwent only LAR 1 year after surgery
2018 Ihnát [35] *N* = 65	LAR only vs NART with LAR	1 year	mRP IniSense UrgeV MaxTV CO	Patients who underwent NART with LAR showed decreased mRP, IniSense, UrgeV, MaxTV, and CO compared to patients who underwent only LAR 1 year after surgery

*NART* neoadjuvant radiotherapy, *LAR* low anterior resection, *mRP* mean resting pressure, *MaxTV* maximum tolerable volume, *MaxRP* maximum resting pressure, *MaxSP* maximum squeeze pressure, *IniSense* initial sensation volume, *UrgeV* urge volume, *CO* compliance

### Correlation between LAR scores and functional ARM in anorectal dysfunction assessment

Postoperative bowel dysfunction occurs in 50–80% of patients who undergo sphincter-preserving surgery for rectal cancer [39]. Therefore, questionnaires evaluating postoperative anorectal sphincter function (patients’ subjective QOL) have continually evolved. One classical tool is the European Organization of Research and Treatment of Cancer Quality of Life Questionnaire (EORTC-QLQ-C30) [40], which encompasses patients’ social and emotional QOL beyond their subjective discomfort. This questionnaire has been used for over 20 years; however, it has shown limitations regarding long-term patient compliance owing to its extensive length. The term low anterior resection syndrome (LARS) was adopted in the 2010s, categorized as follows: (1) frequency of uncontrolled intestinal flatus; (2) fluid incontinence; (3) frequent defecation; (4) clustering; or (5) urgency [41]. The widespread adoption of the term LARS led to the development of the LARS score grading system, with its greatest advantage being its feasibility for patient responses. The LARS score sums up the given scores of the abovementioned five representative symptoms and classifies responders into three categories, “no LARS,” “minor LARS,” and “major LARS,” based on the total score. Systematic reviews have validated this score over the past decade. Despite its simple structure, the LARS score can similarly identify anorectal dysfunction in patients classified as having major LARS, which is conventionally confirmed using longer questionnaires, such as the EORTC-QLQ [42]. Zhang et al. [43] reported that among the 15 clinical studies on LARS in the 2020s, 83% used the LARS score, 13% used the EORTC-QLQC30, and 7% used other questionnaires.

Recent studies using ARM have revealed a trend toward investigating the relationship between numerical manometric data and the LARS score. Confirming the correlation between patients’ questionnaire responses (highly subjective data) and manometry (objective numerical data) is pivotal to facilitating future clinical research. In 2018, Ihnát et al. [44] analyzed the data of 65 patients who underwent LAR and reported a significant relationship between unfavorable manometric parameters (mRP, IniSense, UrgeV, MaxTV, and CO) and an increased LARS score. Despite concerns that the three-tier grading system of the LARS score might not comprehensively classify LARS, the authors reported statistically significant differences in the manometric figures among each of the three LARS score grades. In contrast, Bjoern et al. [45] and Kazi et al. [46] analyzed the relationship between ARM data and LARS score after rectal surgery in 2020 and 2022, respectively, observing no significant differences. These results suggest that the following factors should be considered in studies investigating the relationship between ARM and LARS: (1) ARM and the LARS score should be measured multiple times at consistent intervals owing to the nature of anorectal function recovery over time postoperatively, and (2) the interval between stoma reversal and ARM and LARS measurements should be carefully considered since the diverting stoma introduced during low rectal cancer surgery (for a protective purpose) induces atrophy in anorectal function. In the abovementioned two studies, the time of ARM measurement exceeded 24 months postoperatively. Moreover, Kazi et al. reported that the interval between ileostomy reversal and ARM data measurement should only be 6 months [46]. In 2022, Pi et al. [47] reported data for mid and low rectal cancer at 3 and 6 months postoperatively. In the 3-month data, decreased mRP was associated with “fluid incontinence” and “uncontrolled flatus,” which are subcategories of the LARS score. In the 3- and 6-month data, “low tumor location” was correlated with the “total LARS score” and “urgency.” In the 6-month data, decreased MaxSP was associated with the “total LARS score” and “clustering symptom.” The studies correlating the LARS score and manometric data for anorectal dysfunction assessment are listed in Table 4.

**Table 4:** Recent studies that have examined the correlation between the LARS score and manometric data for anorectal dysfunction assessment

Authors	Object of comparison	F/U interval	Parameters of statistical significance	Interpretation of results
2018 Ihnát [44] *N* = 65	Manometric difference between LARS groups	1 year	mRP IniSense UrgeV MaxTV CO	Patients who underwent LAR and reported “major LARS” scores had decreased mRP, IniSense, UrgeV, MaxTV, and CO compared to patients who underwent LAR and reported “no LARS” scores 1 year after surgery
2020 Bjoern [45] *N* = 36	Manometric difference between LARS groups	3.4 years	No significance	Among patients who received LAR(TATME), there were no significant manometric differences between LARS score groups 3.4 years after surgery
2022 Pi [47] *N* = 24	Correlation between LARS symptoms and manometric parameters	3 months	mRP MaxSP	Among LARS symptoms, “fluid incontinence” and “uncontrolled flatus” were correlated with decreased mRP, whereas “total LARS score” and “clustering” were correlated with decreased MaxSP 3 months after surgery
		6 months	MaxSP	Among LARS symptoms, “total LARS score” and “clustering” were found to be correlated with decreased MaxSP 6 months after surgery

*LARS* low anterior resection syndrome, *mRP* mean resting pressure, *IniSense* initial sensation volume, *UrgeV* urge volume, *MaxTV* maximum tolerable volume, *CO* compliance, *MaxSP* maximum squeeze pressure

The relationship between ARM parameters and LARS severity remains inconsistent. Some studies have reported associations between major LARS and reduced resting pressure, impaired rectal or neorectal sensation, reduced maximum tolerable volume, or decreased compliance, whereas others have failed to demonstrate significant manometric differences between LARS severity groups. These discrepancies may be explained by several factors. First, anorectal function changes dynamically after surgery and stoma reversal; therefore, measurements obtained at 3–6 months may not be comparable with those obtained after 2 years. Second, neoadjuvant radiotherapy, anastomotic height, pelvic fibrosis, and the extent of rectal reservoir loss may affect both symptoms and manometric values. Third, diversion-related disuse changes may alter sphincter and neorectal function, particularly when the interval between stoma reversal and assessment is short. Finally, conventional water-perfused manometry, solid-state manometry, and high-resolution manometry may yield different values and spatial information. Therefore, ARM findings should be interpreted as physiological correlates rather than direct surrogates of LARS severity.

## Future perspectives

The emergence and development of functional ARM have contributed to the physiological assessment of postoperative bowel dysfunction after rectal cancer surgery. Initially a basic research tool, ARM has increasingly been used to characterize anorectal or neo-anorectal physiology after sphincter-preserving procedures, reconstruction, radiotherapy, and stoma reversal. However, its clinical role remains adjunctive, and symptom-based assessment and quality-of-life instruments remain central to the evaluation of rectal cancer survivors.

Unlike conventional manometry, three-dimensional high-resolution manometry can distinguish certain intestinal motilities within the intestinal tract, providing more detailed and useful insights. In 2022, Asnong et al. [48] analyzed the correlation between new motility variables, including the “propagating motor pattern” and “cyclic motor pattern” (available only with three-dimensional high-resolution manometry), and the LARS score. Their analysis showed that the presence of LARS significantly correlates with the “cyclic short antegrade motor pattern,” similar to the pattern observed with the laxative bisacodyl. Other advancements include novel methods, such as measuring intestinal pressure parallel to the intestinal tract’s continuity using tools such as Fecobionics, although full-scale clinical research has not yet been conducted [49, 50].

The diagnosis and grading of low anterior resection syndrome remain primarily clinical and should be based on patient-reported symptoms, validated LARS scores, and quality-of-life questionnaires. ARM should not be regarded as a diagnostic test for LARS or as a stand-alone tool for treatment decision-making after rectal cancer surgery. Rather, its potential value lies in providing adjunctive physiological information in selected clinical or research settings. In postoperative patients, reduced resting pressure may suggest impaired internal anal sphincter function or autonomic nerve injury, whereas reduced squeeze pressure may reflect external sphincter or pelvic floor weakness. Reduced maximum tolerable volume or altered rectal or neorectal sensation may indicate impaired reservoir function, reduced compliance, or hypersensitivity. However, these parameters are not consistently correlated with LARS severity across studies and should be interpreted together with symptom scores, quality-of-life measures, surgical details, radiotherapy exposure, anastomotic height, presence and duration of diversion, and the interval from stoma reversal. At present, there is insufficient evidence to recommend ARM as a routine test to determine the timing of stoma reversal, select a specific treatment for LARS, or predict long-term postoperative bowel function in individual patients. Its role is better defined as a complementary assessment tool for characterizing postoperative anorectal or neo-anorectal physiology, identifying potential mechanisms of dysfunction, documenting baseline function before or after rehabilitation, and improving the design of future clinical studies. ARM may also be useful as an objective physiological outcome measure in studies of pelvic floor rehabilitation. Some researchers have focused on pelvic biofeedback, which has been used for several decades but has lacked sufficient evidence of its effectiveness. In 2021, Sacomori et al. [51] reported a clinical trial protocol termed CARRET to evaluate pelvic rehabilitation using both the LARS score and ARM. This study represents an attempt to assess rehabilitation outcomes by combining patient-reported symptoms with objective physiological measurements.

Despite its clinical utility, ARM presents several challenges in rectal cancer research. Inconsistencies in measurement results and dependency on specific equipment, which vary significantly by manufacturer, constitute major concerns [52]. These issues underscore the necessity for standardizing ARM measurement protocols to achieve uniform results, enhancing its reliability and applicability in clinical practice. Safety concerns are paramount, considering reports [53, 54] of anastomotic perforations during volumetric testing, particularly in patients who have undergone radiotherapy-induced bowel changes. These concerns necessitate a cautious approach to ARM application, emphasizing the need for enhanced safety protocols and patient monitoring.

Future studies should standardize the timing of ARM in relation to surgery and stoma reversal and should stratify or adjust for radiotherapy exposure, anastomotic height, type of reconstruction, pelvic fibrosis, duration of diversion, and the type of manometry system used. Without such standardization, direct comparison of ARM parameters across studies remains difficult. Additionally, future studies may evaluate whether ARM can serve as an objective physiological outcome measure in pelvic floor rehabilitation studies, while recognizing that symptom scores and quality-of-life measures remain the primary clinical outcomes.

## Conclusion

ARM provides objective physiological information on anal sphincter pressure, anorectal reflexes, rectal or neorectal sensation, and reservoir function after rectal cancer surgery. However, postoperative bowel dysfunction, including LARS, remains primarily a clinical diagnosis based on patient-reported symptoms and validated quality-of-life instruments. Current evidence does not support the use of ARM as a stand-alone diagnostic test for LARS or as a routine tool for treatment decision-making, stoma reversal timing, or postoperative counselling. The potential value of ARM lies in its adjunctive role in selected patients and research settings, where it may help characterize mechanisms of dysfunction, document physiological changes over time, and evaluate the effects of rehabilitation strategies.

## Data Availability

No datasets were generated or analysed during the current study.
